# Rate Coefficients of C1 and C2 Criegee Intermediate Reactions with Formic and Acetic Acid Near the Collision Limit: Direct Kinetics Measurements and Atmospheric Implications**

**DOI:** 10.1002/anie.201400964

**Published:** 2014-03-25

**Authors:** Oliver Welz, Arkke J Eskola, Leonid Sheps, Brandon Rotavera, John D Savee, Adam M Scheer, David L Osborn, Douglas Lowe, A Murray Booth, Ping Xiao, M Anwar H Khan, Carl J Percival, Dudley E Shallcross, Craig A Taatjes

**Affiliations:** Combustion Research Facility, Sandia National LaboratoriesStop 9055, Livermore, CA 94551-0969 (USA); The Centre for Atmospheric Science, The School of Earth, Atmospheric and Environmental Science, The University of ManchesterSimon Building, Brunswick Street, Manchester, M13 9PL (UK); Biogeochemistry Research Centre, School of ChemistryThe University of Bristol, Cantock's Close BS8 1TS (UK)

**Keywords:** atmospheric chemistry, Criegee intermediates, gas-phase chemistry, kinetics, organic acids

## Abstract

Rate coefficients are directly determined for the reactions of the Criegee intermediates (CI) CH_2_OO and CH_3_CHOO with the two simplest carboxylic acids, formic acid (HCOOH) and acetic acid (CH_3_COOH), employing two complementary techniques: multiplexed photoionization mass spectrometry and cavity-enhanced broadband ultraviolet absorption spectroscopy. The measured rate coefficients are in excess of 1×10^−10^ cm^3^ s^−1^, several orders of magnitude larger than those suggested from many previous alkene ozonolysis experiments and assumed in atmospheric modeling studies. These results suggest that the reaction with carboxylic acids is a substantially more important loss process for CIs than is presently assumed. Implementing these rate coefficients in global atmospheric models shows that reactions between CI and organic acids make a substantial contribution to removal of these acids in terrestrial equatorial areas and in other regions where high CI concentrations occur such as high northern latitudes, and implies that sources of acids in these areas are larger than previously recognized.

Gas-phase ozonolysis is a major degradation mechanism of alkenes in the Earth’s atmosphere and forms Criegee intermediates (CIs), carbonyl oxides, as reactive intermediates.[1] Many of these CIs have enough energy to decompose, but a fraction becomes collisionally stabilized. These so-called stabilized CIs (in the following simply denoted as CIs, as we consider only the stabilized fraction) can undergo subsequent bimolecular reactions. The chemistry of CIs plays a central role in controlling the budgets of many tropospheric species including OH, organic acids, and secondary organic aerosols (SOA). Until recently all knowledge of the reactivity of CIs had been derived from indirect ozonolysis experiments or theoretical kinetics calculations, and the rate coefficients for reactions of CIs with key atmospheric species have been uncertain by several orders of magnitude.[2] The discovery that CIs are formed in the reactions of α-iodoalkyl radicals with O_2_[3] has enabled direct kinetics measurements of CI reactions.[3,4] These measurements revealed that the rate coefficients of CI reactions with several key atmospheric species are substantially different than models had assumed.[2]

The reaction of CIs with organic acids can provide a pathway in which alkenes are converted to low-volatility compounds and thus contribute to the formation of SOA. Here we report the direct determination of the rate coefficients for the reactions of two CIs, CH_2_OO and both conformers[4d] of CH_3_CHOO, with formic acid (HCOOH) and acetic acid (CH_3_COOH) (reactions (1)–(6)).

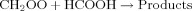
(1)

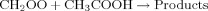
(2)

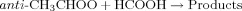
(3)

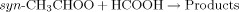
(4)

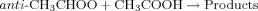
(5)

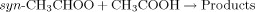
(6)

Two complementary techniques are employed for time-resolved detection of the CIs: multiplexed photoionization mass spectrometry (MPIMS)[3,4d] (for CH_2_OO, *syn*- and *anti*-CH_3_CHOO) and cavity-enhanced broadband ultraviolet (UV) absorption spectroscopy[4a] (for CH_2_OO). The measured rate coefficients are in excess of 1×10^−10^ cm^3^ s^−1^, several orders of magnitude faster than results from experiments of alkene ozonolysis in the presence of carboxylic acids[5] and what modeling studies have employed.[6] With these rate coefficients, global atmospheric modeling and steady-state analysis shows that CI reactions with organic acids have significant effects on concentrations of acids and CI in the environment.

Figure 1 a shows time profiles of CH_2_OO from the MPIMS experiments, and Figure 2 a shows a corresponding plot for CH_2_OO from the UV absorption experiments. In the absence of added co-reactant, the CI signals follow a first-order decay, *k*_loss_, that reflects both loss on the walls (dominant under these low-pressure conditions), and unimolecular decomposition. Upon adding acid, the decay of CI becomes faster. The rate coefficients for reactions (1)–(6) were determined by measuring the CI decay as a function of the acid concentration. Time profiles of the CIs were fitted to a single exponential decay, convolved with the temporal instrument response function.[3,4] The rate coefficient for the pseudo-first-order loss of the CI depends linearly on [acid], with the slope yielding the second-order rate coefficient for the CI reaction with acid and the intercept reflecting *k*_loss_. Such plots are shown in Figure 1 b for reactions (1), (3), and (4), in Figure 2 b for reaction (2), and for the other reactions in Figures S1 and S2 (see the Supporting Information).

**Figure 1:**
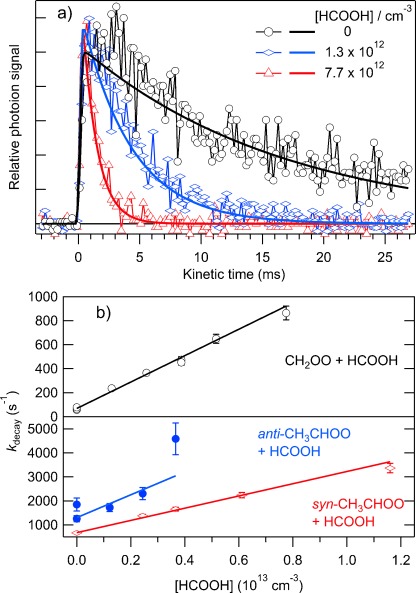
Kinetics results from the MPIMS experiments. a) CH_2_OO time traces as a function of [HCOOH] taken using a H_2_ discharge lamp for ionization, and fits to the experimental data. b) Dependence of the decay constant of CH_2_OO (black; taken with a H_2_ discharge), *anti*-CH_3_CHOO (blue; taken using 9.35 eV synchrotron radiation), and *syn*-CH_3_CHOO (blue; taken using 10.5 eV synchrotron radiation). The solid lines are fits to the experimental data. The slopes are the second-order rate coefficients for the CI+acid reactions. Error bars reflect the 1σ uncertainties from the fits of the experimental time profiles.

**Figure 2:**
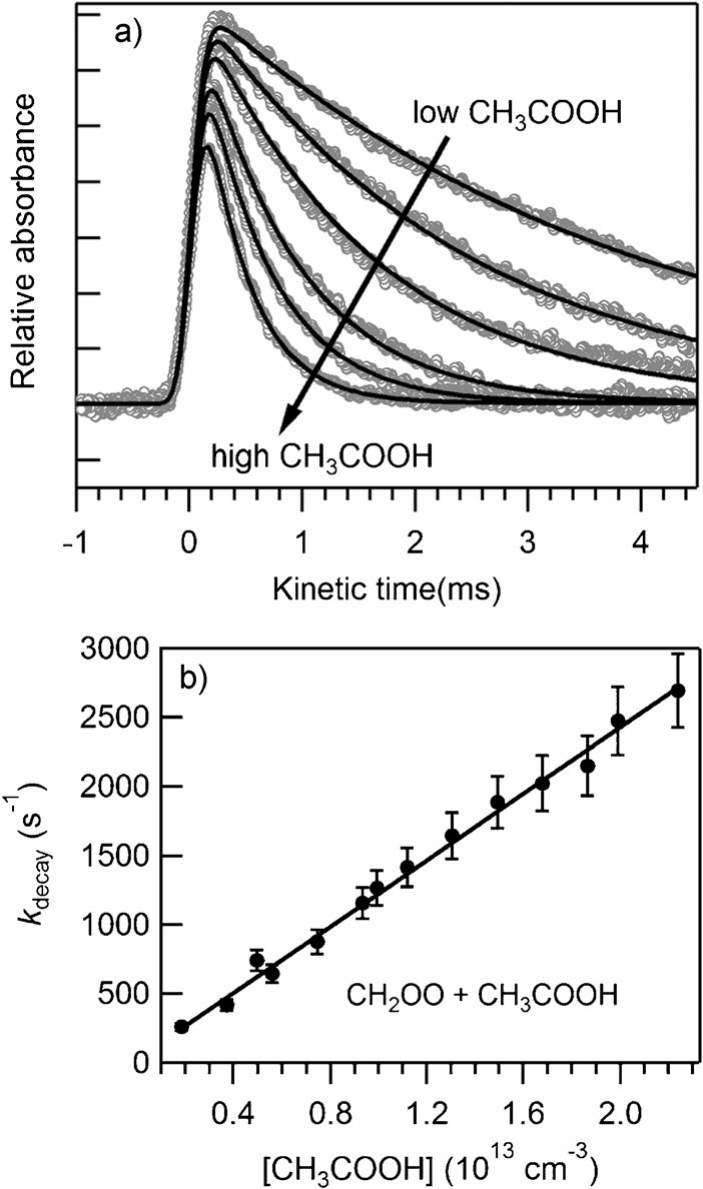
Kinetics results from the UV absorption experiments. a) CH_2_OO time traces for [CH_3_COOH] in the range (1.86–22.39)×10^12^ cm^−3^, and fits to the experimental data. b) Dependence of the decay constant of CH_2_OO on [CH_3_COOH]. The solid line is a fit to the experimental data with the slope as the second-order rate coefficient for the CH_2_OO+CH_3_COOH reaction. Error bars shown are 15 % of the nominal value as estimated from repeated experiments at identical conditions.

Table 1 summarizes the rate coefficients for reactions (1)–(6) determined in this work. The values for the CH_2_OO reactions obtained with the two independent experimental techniques are in close agreement. Furthermore, the rate coefficient does not decrease with increasing size of either the CI or the carboxylic acid, suggesting that the rate coefficients of other CI+carboxylic acid reactions are also large. We find negligible conformer dependence in the rate coefficients for the *anti*- and *syn*-CH_3_CHOO reactions.

**Table 1:** Rate coefficients for CI+carboxylic acid reactions determined in this work at 298 K. Error bounds represent 95 % confidence interval.

	Reaction	*k* [×10^−10^ cm^3^ s^−1^]	Method	*p* [Torr]
(1)	CH_2_OO+HCOOH	1.1±0.1	MPIMS	4
		1.1±0.1	UV	5
(2)	CH_2_OO+CH_3_COOH	1.3±0.1	MPIMS	4
		1.2±0.1	UV	5
(3)	*anti*-CH_3_CHOO+HCOOH	5±3	MPIMS	4
(4)	*syn*-CH_3_CHOO+HCOOH	2.5±0.3	MPIMS	4
(5)	*anti*-CH_3_CHOO+CH_3_COOH	2.5±0.6	MPIMS	4
(6)	*syn*-CH_3_CHOO+CH_3_COOH	1.7±0.5	MPIMS	4

No direct measurements of CI+carboxylic acid reactions exist in the literature. However, Neeb et al.[7] derived a ratio of *k*_1_ to the rate coefficient for Reaction (7)

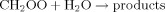
(7)

of *k*_1_/*k*_7_≈14 000 at 293 K and 730 Torr from ethylene ozonolysis experiments in the presence of water vapor. Taking *k*_7_<9×10^−17^ cm^3^ s^−1^,[4b] this ratio would yield *k*_1_<1.3×10^−12^ cm^3^ s^−1^. By adding acetic acid to the O_3_/2-methylbut-2-ene system Johnson et al.[5a] derived *k*(CI+CH_3_COOH)<1×10^−14^ cm^3^ s^−1^. The present rate coefficients are more than two orders of magnitude larger than these literature upper limits. However, recent ozonolysis measurements[8] show CI reactions with acids to be about three times faster than the rapid[3,4d] reaction with SO_2_. Quantum-chemical studies suggest that the CH_2_OO+HCOOH reaction proceeds through barrierless association to hydroperoxymethyl formate (HPMF, HC(O)OCH_2_OOH), with no stable “pre-reactive” complex identified.[9] The slower CI reactions with aldehydes and ketones,[4c] do exhibit reactant-channel van der Waals complexes that may affect the overall kinetics.[10] The large CI+acid rate coefficients corroborate the absence of an entrance barrier and suggest that the bottlenecks to reaction of CI with carbonyl groups are absent in reaction of CI with acids. Facile transfer of the acidic -OH hydrogen to the basic terminal O atom of the CI, forming the hydroperoxyalkyl carboxylate (e.g., HPMF), may act to prevent any kinetically significant entrance-channel complex.

Reliable kinetics information for individual CI reactions can be difficult to obtain from ozonolysis experiments owing to its inherent complexity,[2] and large removal rate coefficients in the direct determinations suggest reinterpretation of earlier ozonolysis measurements.[5a,^7^,^11^] However, the consequences of the reaction of CI with acids in indirect ozonolysis experiments will depend on the products of the reactions. Ozonolysis experiments by Neeb et al.[11] found evidence that the CH_2_OO+HCOOH reaction forms HPMF, which subsequently dehydrates to formic anhydride (FAN, (CHO)_2_O). As discussed in the Supporting Information, no significant product signal is detected at the parent masses of HPMF (*m*/*z*=92) and FAN (*m*/*z*=74). However, signal observed at *m*/*z*=64 and *m*/*z*=31 might arise from dissociative ionization of HPMF or from hydroperoxymethanol (HOCH_2_OOH, *m*/*z*=64). For reactions (2)–(6), no significant product signal appears at the parent masses of the CI–acid adducts, although ion signals are observed that may arise from dissociative ionization of these adducts. Further work is necessary to characterize products from the CI+acids reactions.

The large rate coefficients determined here suggest that CIs could be important reaction partners for organic acids, and that reaction with organic acids could compete with unimolecular decay and reaction with water as key loss processes for CIs under some conditions. A simple comparison of loss rates (Table S2) considers CH_2_OO+H_2_O [R (7)] alongside Reactions (8) and (9).

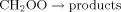
(8)

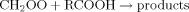
(9)

Assuming values for *k*_7_ of 1×10^−16^ cm^3^ s^−1^ or 1×10^−17^ cm^3^ s^−1^,[2,4b] setting *k*_8_ to 100 s^−1^, and applying values of *k*_9_ of 1×10^−10^ cm^3^ s^−1^ or 5×10^−10^ cm^3^ s^−1^ suggests that removal by organic acids (RCOOH) will become significant for CIs at acid levels of around 1 ppb (Table S2). Such levels have been observed in forest environments,[12] in urban outflow,[13] and also in urban environments,[14] where a direct emission source from vehicles has been inferred.

The analysis of Vereecken et al.,[6] which employs smaller estimates of *k*_8_, predicted CH_2_OO and *anti*-CH_3_CHOO to be principally consumed by reactions with H_2_O or (H_2_O)_2_. For the reaction of CIs with acids, Vereecken et al.[6] assumed a lumped rate coefficient of 5×10^−12^ cm^3^ s^−1^. Using *k*_1_=1.1×10^−10^ cm^3^ s^−1^ as a proxy for all CH_2_OO+acids reactions, and using the most recent rate coefficients[4b] for the reaction with water, *k*_7_<9×10^−17^ cm^3^ s^−1^ (cf. 5.6×10^−15^ cm^3^ s^−1^ in Ref. [6]) and with the water dimer, *k*(CH_2_OO+(H_2_O)_2_)<3×10^−13^ cm^3^ s^−1^ (deduced from Ref. [4b] and the 2 H_2_O⇌(H_2_O)_2_ equilibrium constant; cf. 1×10^−10^ cm^3^ s^−1^ in Ref. [6]), predicts that in the boreal forest environment 10 % of CH_2_OO react with acids. The impact of acid reactions would be relatively larger for *syn*-CH_3_CHOO, which reacts much more slowly with water.[15] The present results suggest that all rate coefficients for reaction of a CI with a carboxylic acid are on the order of 1×10^−10^ cm^3^ s^−1^. If so, it is very likely that reaction with acids will be a significant loss process for larger CIs.

The effect of these reactions on the acid concentrations is shown by surface HCOOH fields derived from a global model integration, assuming an even more conservative *k*_8_=150 s^−1^ (Figure 3). The reaction with CIs contributes substantially to organic acid removal in equatorial regions, and also in some northern high latitude locations (where the model generates elevated terpene levels). This model is too coarse to interrogate urban environments, where there may also be elevated levels of CIs[16] and hence a substantial reduction in organic acid lifetime. The indoor environment is another region where CI reactions with organic acids may be important;[2] recent estimates[17] suggest that peak indoor CI levels could be in the region of 10^5^ or even 10^6^ cm^−3^.

**Figure 3:**
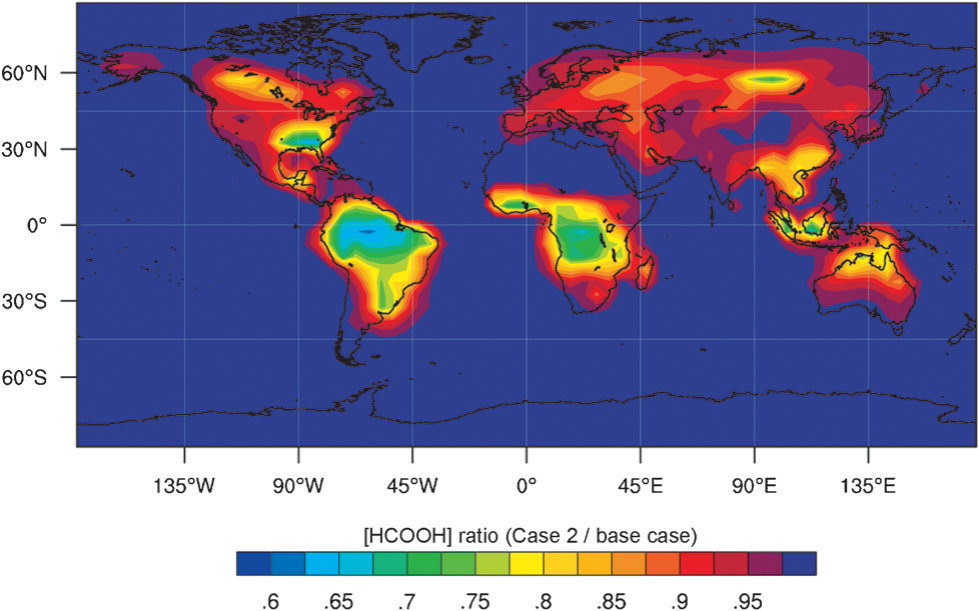
Ratio of formic acid concentration predicted using the present rate coefficients for reaction of CIs with organic acids (case 2, Table S1) to the concentration from a base model neglecting CI reactions with acids.

Reactions of CIs with acids may lead to adduct formation; preliminary calculations suggest that these adducts may have lower vapor pressures than the reactants, as shown in Table S3. If the products are more condensable than the parent acid these reactions could contribute to SOA formation. Quantification of reaction products and rate coefficients as a function of the structure are needed before the impact of CI reactions with carboxylic acids on SOA can be fully assessed. Nevertheless, the increased removal rates indicated by the present direct kinetics determinations imply that environmental sources of organic acids must be correspondingly larger to agree with field measurements.
