# Supplementary material for: Rate Coefficients of C1 and C2 Criegee Intermediate Reactions with Formic and Acetic Acid Near the Collision Limit: Direct Kinetics Measurements and Atmospheric Implications
Source: Angew Chem Int Ed Engl. 2014 Mar 25;53(18):4547–50. doi: 10.1002/anie.201400964 (PMC4499262; doi:10.1002/anie.201400964)
Supplement: Supplementary file 1 [file anie0053-4547-sd1.pdf]

Angewandte Chemie
Eine Zeitschrift der Gesellschaft Deutscher Chemiker

Supporting Information

© Wiley-VCH 2014

69451 Weinheim, Germany

Rate Coefficients of C1 and C2 Criegee Intermediate Reactions with Formic and Acetic Acid Near the Collision Limit: Direct Kinetics Measurements and Atmospheric Implications**

Oliver Welz, Arkke J. Eskola, Leonid Sheps, Brandon Rotavera, John D. Savee, Adam M. Scheer, David L. Osborn, Douglas Lowe, A. Murray Booth, Ping Xiao, M. Anwar H. Khan, Carl J. Percival, Dudley E. Shallcross,* and Craig A. Taatjes**

anie_201400964_sm_miscellaneous_information.pdf

Supplementary information

Experimental and Modeling Details

Experiment. Pulsed-laser photolysis of a diiodoalkane precursor (CH_2I_2 or CH_3CHI_2) at 351 nm or 266 nm forms α-iodoalkyl radicals, which subsequently react with O_2 and form CIs. The HCOOH or CH_3COOH reactants are introduced from dilute premixes, whose concentration is calculated accounting for dimerization.[1] MPIMS experiments (298 K and 4 Torr) used the Sandia multiplexed chemical kinetics reactor, as in previous kinetics studies of CH_2OO[2] and CH_3CHOO[3] reactions. The reacting mixture is analyzed using time-resolved photoionization mass spectrometry with vacuum ultraviolet (VUV) photoionization. The VUV source was either a H_2 discharge (principally Lyman-a (10.2 eV)) or beamline 9.0.2 of the Advanced Light Source providing tunable synchrotron radiation. Conformer-dependent kinetics of CH_3CHOO was probed at 9.35 eV and 10.5 eV: at 9.35 eV, only the anti-conformer is ionized, whereas at 10.5 eV signal is dominated by syn-CH_3CHOO.[3]

Direct UV absorption probing of CH_2OO reactions with HCOOH and CH_3COOH (298 K and 5 Torr) took place in a time-resolved broadband cavity-enhanced absorption spectrometer.[4] The apparatus consists of an uncoated slow-flow quartz reactor, integrated into a broadband optical resonator cavity, operated over the 300 – 450 nm wavelength range. Here 266-nm photolysis was chosen to reduce scattered-laser interference in the probe spectral region. A Xe arc lamp provides continuous probe radiation, and the average effective path length of the pump-probe overlap region is ~50 m over the probe wavelength range. A custom-built spectrometer (2 nm resolution (FWHM)) acquires the complete temporal evolution (120 μs resolution) of the full absorption spectrum for each laser shot, averaged for 120 - 540 shots. Transient absorption is obtained from the difference between photolysis ON and photolysis OFF spectra.

Initial number densities (in cm^{-3}) for the data in Figure 1 were $[O_2] = 1.0 \times 10^{16}$ and $[CH_2I_2] = 1.7 \times 10^{13}$, $[CH_2OO] \sim 4 \times 10^{11}$ or $[CH_3CHI_2] = 4.0 \times 10^{13}$, $[CH_3CHOO] \sim 1 \times 10^{12}$. Initial number densities (in cm^{-3}) for the data in Figure 2 were: $[CH_2I_2] = 3.2 \times 10^{13}$, $[CH_2OO] \sim 5 \times 10^{11}$, and $[O_2] = 8.2 \times 10^{15}$.

Model. The STOCHEM-CRI global atmospheric model has been described in detail elsewhere.[5] In the integrations presented here, reactions between the CIs CH_2OO, CH_3CHOO and larger CIs (≥ C3) with organic acids have been included, although for simplicity only the HCOOH fields are presented. Results are calculated for a base case[5b] that treats CI reactions with acids as negligible (case 1) and two scenarios using the present rate coefficients and two assumptions for reactions of larger CIs: Case 2, k(other CI + HCOOH) = 1.1×10^{-10} cm^3 s^{1}; Case 3, k(other CI + HCOOH) = 2.5×10^{-10} cm^3 s^{1} (see Table S1).

Table S1: Rate coefficients for CI reactions in the model scenarios

	**Base scenario (case 1)**	**Case 2**	**Case 3**
**CI + H_2O**	1×10^{-16} cm^3 s^{-1}	1×10^{-16} cm^3 s^{-1}	1×10^{-16} cm^3 s^{-1}
**CI unimolecular loss**	150 s^{-1}	150 s^{-1}	150 s^{-1}
**CH_2OO + acids**	–	1.1×10^{-10} cm^3 s^{-1}	1.1×10^{-10} cm^3 s^{-1}
**CH_3COO + acids**	–	2.5×10^{-10} cm^3 s^{-1}	2.5×10^{-10} cm^3 s^{-1}
**Other CI + acids**	–	1.1×10^{-10} cm^3 s^{-1}	2.5×10^{-10} cm^3 s^{-1}

Table S2: Percentage loss rates for Criegee intermediates via reactions (7-9) using k_8 = 100 s^{-1} and a variety of other assumed rate coefficients, at two temperatures and two [H_2O].

Temp./K	k / cm^3 $molecule^{-1}$ s^{-1}	RH	[H_2O] cm^{-3}	[Acid] ppb	% loss rxn. 7	% loss rxn. 8	% loss rxn. 9
283	$k_7 = 1 \times 10^{-16}$ $k_9 = 1 \times 10^{-10}$	50%	1.57×10^{17}	1	13.3	84.6	2.1
283	$k_7 = 1 \times 10^{-17}$ $k_9 = 1 \times 10^{-10}$	50%	1.57×10^{17}	1	1.5	96.1	2.4
283	$k_7 = 1 \times 10^{-16}$ $k_9 = 1 \times 10^{-10}$	100%	3.14×10^{17}	1	23.5	74.7	1.8
283	$k_7 = 1 \times 10^{-17}$ $k_9 = 1 \times 10^{-10}$	100%	3.14×10^{17}	1	3.0	94.7	2.3
298	$k_7 = 1 \times 10^{-16}$ $k_9 = 1 \times 10^{-10}$	50%	3.85×10^{17}	1	27.3	70.9	1.8
298	$k_7 = 1 \times 10^{-17}$ $k_9 = 1 \times 10^{-10}$	50%	3.85×10^{17}	1	3.6	94.0	2.4
298	$k_7 = 1 \times 10^{-16}$ $k_9 = 1 \times 10^{-10}$	100%	7.7×10^{17}	1	42.9	55.7	1.4
298	$k_7 = 1 \times 10^{-17}$ $k_9 = 1 \times 10^{-10}$	100%	7.7×10^{17}	1	7.0	90.7	2.3
298	$k_7 = 1 \times 10^{-16}$ $k_9 = 1 \times 10^{-10}$	100%	7.7×10^{17}	10	40.6	52.8	6.6
298	$k_7 = 1 \times 10^{-17}$ $k_9 = 5 \times 10^{-10}$	100%	7.7×10^{17}	10	6.4	83.2	10.4

Table S3. A summary of the calculated vapor pressures for assumed products of CI reactions with organic acids. Vapor pressures were calculated using the Nannoolal et al.[6] vapor pressure estimation method. Vapor pressures above 4.0 Pa will not partition to the aerosol phase.[7]

	Vapor pressure (Pa)			
R group (carbon chain length)	Adduct product from RCOOH + CH_2OO	Adduct product from RCOOH + CH_3CHOO	Ring opening product from RCOOH + CH_2OO	Ring opening product from RCOOH + CH_3CHOO
0	7170.0	5281.6	214.3	132.1
1	3639.1	2691.5	126.2	76.5
2	1067.4	815.0	39.5	24.1
3	321.6	252.9	12.5	7.7
4	99.2	80.1	4.0	2.5
5	31.2	25.8	1.3	0.8
6	10.0	8.4	0.4	0.3
7	3.2	2.8	0.1	0.1

Products of the CH_2OO + HCOOH reaction

The synchrotron photoionization spectra show product species that produce ions at *m*/*z* = 31 (CH_3O, appearance energy (AE) ~ 10.8 eV) and *m*/*z* = 64 (CH_4O_3, AE ~ 10.3 eV). These product signals were observed only when HCOOH was present. No product signal appears at the parent mass of a CH_2OO-HCOOH adduct (*m*/*z* = 92) or FAN (*m*/*z* = 74). Because the photoionization spectra of HPMF and FAN are unknown, the experiments do not necessarily rule out their formation, particularly if low-lying dissociative ionization channels exist. CBS-QB3 calculations[8] using Gaussian09[9] place the adiabatic ionization energy (AIE) of HPMF at 10.22 eV. As this energy is lower than the AEs of the *m*/*z* = 31 and *m*/*z* = 64 signals, it is possible that the *m*/*z* = 31 and *m*/*z* = 64 cations arise from dissociative ionization of HPMF. The chemical formula of FAN ($C_2H_2O_3$) rules out formation of the *m*/*z* = 31 (CH_3O) and *m*/*z* = 64 (CH_4O_3) signals via dissociative ionization of FAN; no evidence of FAN formation is observed under the present conditions.

The calculated AIE of hydroperoxymethanol (HPM, $HOOCH_2OH$), another plausible product at *m*/*z* = 64, is 9.90 eV, lower than the 10.3 eV observed AE. However, the HPM cation has an elongated C–OOH bond (1.61 Å vs 1.40 Å in the neutral), which could cause the apparent onset to be higher than the calculated AIE. Moreover, the thermodynamic appearance energy is 10.76 eV for the CH_2OH fragment ion from HPM (corresponding to HO_2 loss), similar to the ~ 10.8 eV onset observed for the *m*/*z* = 31 product signal. As a consequence, the observed product spectrum is consistent with HPM product as well as with a stabilized CH_2OO-HCOOH adduct, possibly HPMF. Further analysis of the products from the CH_2OO + HCOOH reaction would require the photoionization spectra for parent and fragment ions of potential products, which is outside the scope of the present study.

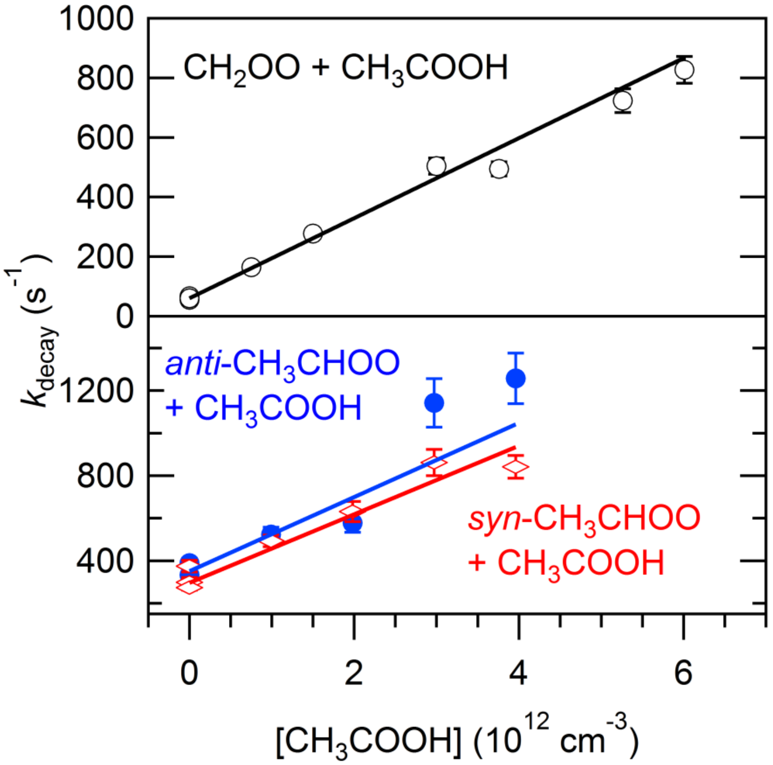

Figure S1: Kinetics results from the MPIMS experiments for C.I. reactions with acetic acid. Dependence of the decay constant of CH_2OO (black; taken with a H_2 discharge), *anti*-CH_3CHOO (blue; taken using 9.35 eV synchrotron radiation), and *syn*-CH_3CHOO (red; taken using 10.5 eV synchrotron radiation) on [CH_3COOH]. The solid lines are fits to the experimental data. The slopes are the second order rate coefficients for the C.I. + acetic acid reactions. Error bars shown are the 1σ uncertainties from the fits of the experimental time profiles. Initial number densities (in cm^{-3}) were [O_2] = 1.0×10^{16} and [CH_2I_2] = 1.6×10^{13}, [CH_2OO] ~ 4×10^{11} or [CH_3CHI_2] = 3.0×10^{13}, [CH_3CHOO] ~ 7×10^{11}.

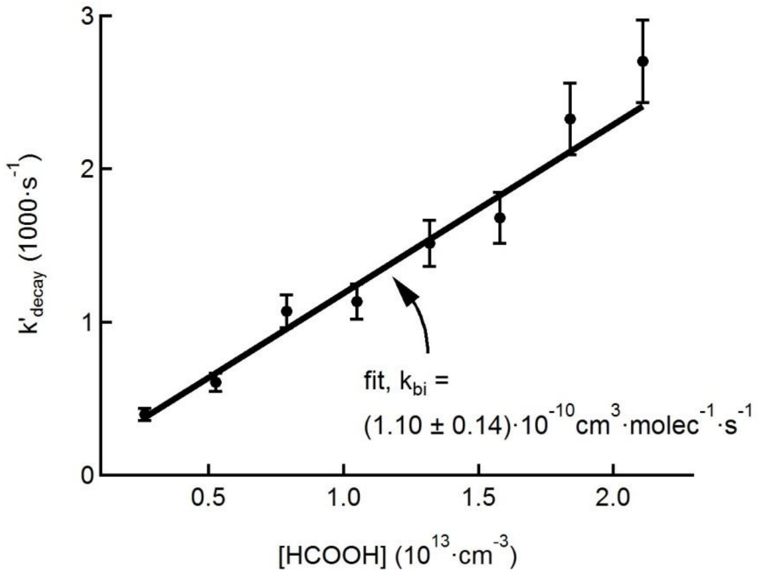

Figure S2: Kinetics results from the UV absorption experiments for the CH_2OO + HCOOH reaction at 295 K and 5.1 Torr: Dependence of the decay constant of CH_2OO on [HCOOH] (dots). The solid line is a fit to the experimental data with the slope as the second order rate coefficient for the CH_2OO + CH_3COOH reaction. Error bars shown are 15% of the nominal value as estimated from repeated experiments at identical conditions. Initial number densities (in cm^{-3}) were: $[CH_2I_2] = 3.2 \times 10^{13}$, $[CH_2OO] \sim 5 \times 10^{11}$, and $[O_2] = 8.6 \times 10^{15}$.

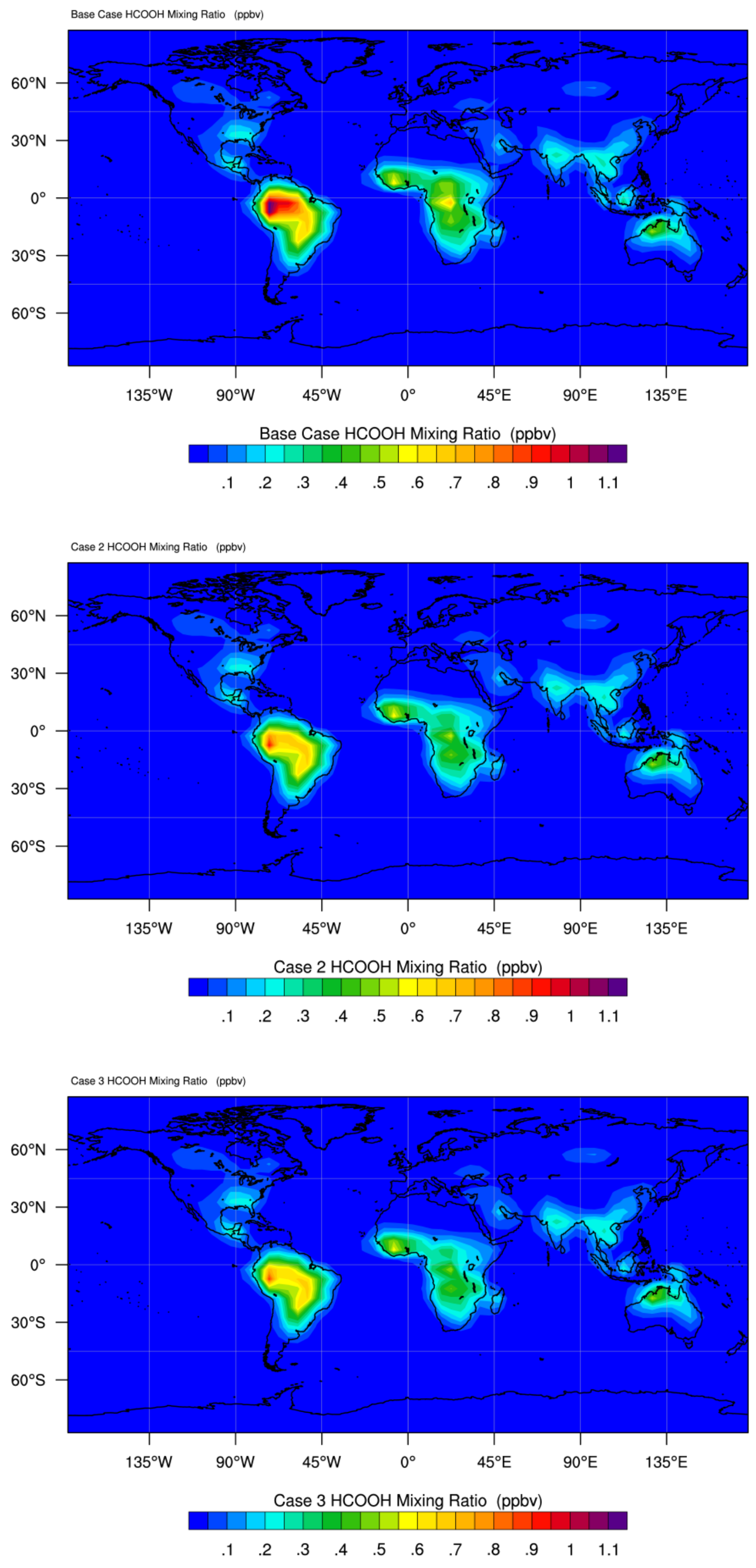

Figure S3. Absolute modeled mixing ratios for formic acid under the three scenarios (Table S1).

Supplementary References

[1] A. Winkler, P. Hess, *J. Am. Chem. Soc.* **1994**, *116*, 9233-9240.

[2] a) C. A. Taatjes, O. Welz, A. J. Eskola, J. D. Savee, D. L. Osborn, E. P. F. Lee, J. M. Dyke, D. W. K. Mok, D. E. Shallcross, C. J. Percival, *Phys. Chem. Chem. Phys.* **2012**; b) O. Welz, J. D. Savee, D. L. Osborn, S. S. Vasu, C. J. Percival, D. E. Shallcross, C. A. Taatjes, *Science* **2012**, *335*, 204-207.

[3] C. A. Taatjes, O. Welz, A. J. Eskola, J. D. Savee, A. M. Scheer, D. E. Shallcross, B. Rotavera, E. P. F. Lee, J. M. Dyke, D. K. W. Mok, D. L. Osborn, C. J. Percival, *Science* **2013**, *340*, 171-180.

[4] a) L. Sheps, *J. Phys. Chem. Lett.* **2013**, 4201-4205; bL. Sheps, D. W. Chandler, Sandia National Laboratories, **2013**, p. 26.

[5] a) A. T. Archibald, M. C. Cooke, S. R. Utembe, D. E. Shallcross, R. G. Derwent, M. E. Jenkin, *Atmos. Chem. Phys.* **2010**, *10*, 8097-8118; b) C. Percival, O. Welz, A. J. Eskola, J. D. Savee, D. L. Osborn, D. O. Topping, D. Lowe, S. Utembe, A. Bacak, G. McFiggans, M. Cooke, A. T. Archibald, M. Jenkin, R. G. Derwent, I. Riipinen, D. Mok, E. P. F. Lee, J. Dyke, C. A. Taatjes, D. E. Shallcross, *Faraday Discuss.* **2013**, *165*, 45-73 ; c) S. R. Utembe, M.C.Cooke, A. T. Archibald, M. E. Jenkin, R. G. Derwent, D. E. Shallcross, *Atmos. Environ.* **2010**, *44*, 1609-1622.

[6] a) Y. Nannoolal, J. Rarey, D. Ramjugernath, *Fluid Phase Equilibr.* **2008**, *269*, 117-133; b) Y. Nannoolal, J. Rarey, D. Ramjugernath, W. Cordes, *Fluid Phase Equilibr.* **2004**, *226*, 45-63.

[7] a) M. H. Barley, G. McFiggans, *Atmos. Chem. Phys.* **2010**, *10*, 749-767; b) A. M. Booth, M. H. Barley, D. O. Topping, G. Mcfiggans, A. Garforth, C. J. Percival, *Atmos. Chem. Phys.* **2010**, *10*, 4879-4892.

[8] a) J. A. Montgomery, M. J. Frisch, J. W. Ochterski, G. A. Petersson, *J. Chem. Phys.* **1999**, *110*, 2822-2827; b) J. A. Montgomery, M. J. Frisch, J. W. Ochterski, G. A. Petersson, *J. Chem. Phys.* **2000**, *112*, 6532-6542.

[9] M. J. Frisch, G. W. Trucks, H. B. Schlegel, G. E. Scuseria, M. A. Robb, J. R. Cheeseman, G. Scalmani, V. Barone, B. Mennucci, G. A. Petersson, H. Nakatsuji, M. Caricato, X. Li, H. P. Hratchian, A. F. Izmaylov, J. Bloino, G. Zheng, J. L. Sonnenberg, M. Hada, M. Ehara, K. Toyota, R. Fukuda, J. Hasegawa, M. Ishida, T. Nakajima, Y. Honda, O. Kitao, H. Nakai, T. Vreven, J. Montgomery, J. A., J. E. Peralta, F. Ogliaro, M. Bearpark, J. J. Heyd, E. Brothers, K. N. Kudin, V. N. Staroverov, R. Kobayashi, J. Normand, K. Raghavachari, A. Rendell, J. C. Burant, S. S. Iyengar, J. Tomasi, M. Cossi, N. Rega, N. J. Millam, M. Klene, J. E. Knox, J. B. Cross, V. Bakken, C. Adamo, J. Jaramillo, R. Gomperts, R. E. Stratmann, O. Yazyev, A. J. Austin, R. Cammi, C. Pomelli, J. W. Ochterski, R. L. Martin, K. Morokuma, V. G. Zakrzewski, G. A. Voth, P. Salvador, J. J. Dannenberg, S. Dapprich, A. D. Daniels, Ö. Farkas, J. B. Foresman, J. V. Ortiz, J. Cioslowski, D. J. Fox, Revision A.1 ed., Gaussian, Inc, Wallingford CT, **2009**.
